# Supplementary material for: Similar neural responses predict friendship
Source: Nat Commun. 2018 Jan 30;9:332. doi: 10.1038/s41467-017-02722-7 (PMC5790806; doi:10.1038/s41467-017-02722-7)
Supplement: Supplementary file 1 — Supplementary Information [file 41467_2017_2722_MOESM1_ESM.pdf]

Supplementary Figures

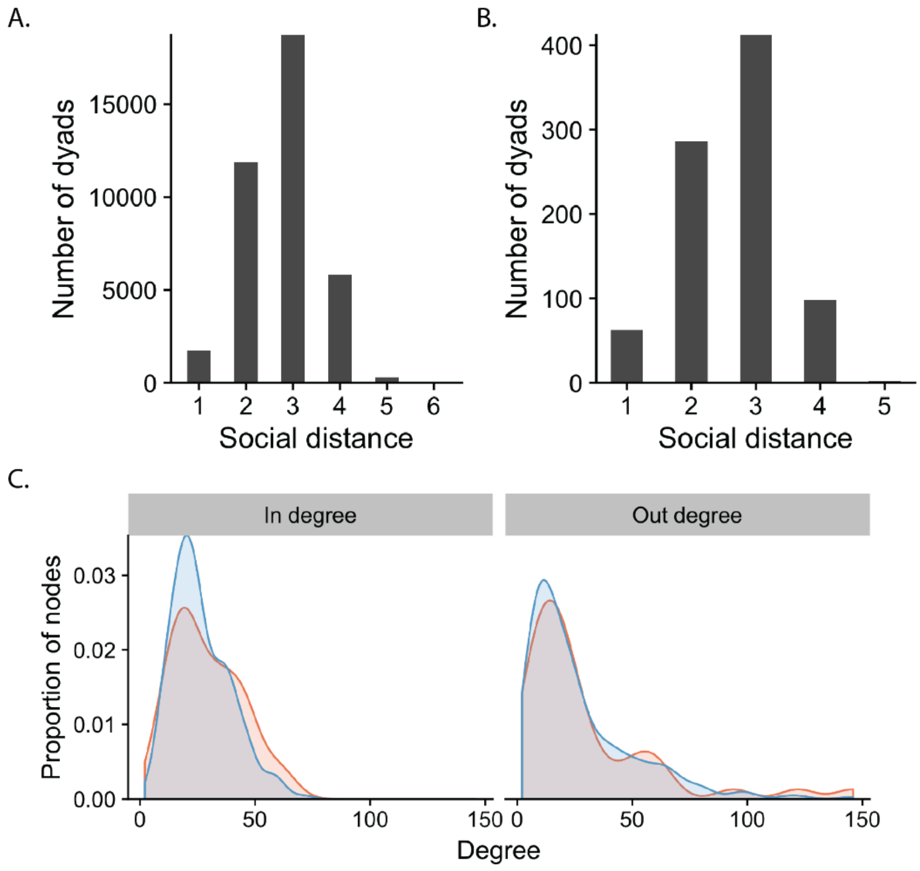

Supplementary Fig. 1. Degree and social distance distributions. (A) Distribution of shortest paths (comprised of mutually reported social ties) between students in the entire academic cohort (279 participants; 38,781 unique dyads) and **(B)** between students who participated in the fMRI study (42 participants; 861 unique dyads). **(C)** Degree distributions within the fMRI sample (N = 42) and within the entire academic cohort (N = 279) are shown in orange and blue, respectively.

Supplementary Tables

Supplementary Table 1. Summary of participants' prior familiarity with the video clips used in the fMRI study

Clip	Title	Number of fMRI participants who had seen clip before (/42)	Number of dyads of fMRI participants who had both seen clip before (/861)
1	'An Astronaut's View of Earth'	0	0
2	Google Glass review	1	0
3	'Crossfire'	0	0
4	'All I Want'	2	1
5	Wedding film	0	0
6	Scientific demonstration	3	3
7	'Food Inc.'	0	0
8	'We Can Be Heroes'	1	0
9	'Ban College Football'	0	0
10	Soccer match	1	0
11	Baby sloth sanctuary	2	1
12	'Ew!'	3	3
13	'Life's Too Short'	4	6
14	'America's Funniest Home Videos'	0	0

Supplementary Table 2. Frequency of missing data for each anatomical ROI

	Number of subjects missing data (/42)	
	**Left**	**Right**
Temporal lobe – medial aspect		
Entorhinal area	3	3
Parahippocampal gyrus	1	2
Temporal pole	2	2
Fusiform gyrus	2	2
Temporal pole – lateral aspect		
Superior temporal gyrus	0	0
Middle temporal gyrus	1	0
Inferior temporal gyrus	1	2
Transverse temporal gyrus	0	0
Banks of the superior temporal sulcus	1	0
Frontal lobe		
Superior frontal gyrus	0	0
Posterior middle frontal gyrus	0	0
Anterior middle frontal gyrus	0	0
Inferior frontal gyrus, *pars opercularis*	0	0
Inferior frontal gyrus, *pars triangularis*	0	0
Inferior frontal gyrus, *pars orbitalis*	0	0
Lateral orbital gyrus	0	0
Medial orbital gyrus	0	0
Frontal pole	0	0
Precentral gyrus	0	0
Paracentral lobule	0	0
Parietal lobe		
Postcentral gyrus	0	0
Supramarginal gyrus	0	0
Superior parietal cortex	0	0
Inferior parietal cortex	0	0
Precuneus	0	0
Occipital Lobe		
Lingual gyrus	1	1
Pericalcarine cortex	1	1
Cuneus	0	0
Lateral occipital gyrus	1	1
Cingulate cortex		
Rostral anterior cingulate gyrus	0	0
Caudal anterior cingulate gyrus	0	0
Posterior cingulate gyrus	0	0
Isthmus of the cingulate gyrus	0	0
Insula and subcortical structures		
Insula	0	0
Amygdala	1	0
Caudate nucleus	0	0
Hippocampus	1	1
Globus pallidus	0	0
Putamen	0	0
Nucleus accumbens	0	0

**Supplementary Table 3.** Predicting social distance based on weighted average neural similarities and dyadic differences in control variables (ordered logistic regression analyses with multi-way clustering for each video)				
**Predictor**	***ß***	***SE***	***t***	***p*-value**
**Comedic videos**				
**"Ew!"**				
Neural similarity***	-0.75	0.22	-3.42	0.00066
Handedness	0.08	0.06	1.36	0.17
Nationality***	0.54	0.15	3.61	0.00032
Gender**	0.38	0.12	3.27	0.0011
Ethnicity	0.06	0.09	0.71	0.48
Age	0.15	0.14	1.08	0.28
**"America's Funniest Home Videos"**				
Neural similarity	0.39	0.25	1.58	0.12
Handedness	0.07	0.06	1.21	0.22
Nationality***	0.62	0.15	4.00	0.000069
Gender**	0.38	0.12	3.15	0.0017
Ethnicity	0.09	0.09	0.94	0.35
Age	0.15	0.14	1.06	0.29
**"We Can Be Heroes"**				
Neural similarity*	-0.48	0.21	-2.32	0.020
Handedness	0.09	0.06	1.48	0.14
Nationality***	0.56	0.15	3.74	0.00019
Gender**	0.38	0.12	3.18	0.0015
Ethnicity	0.09	0.09	0.95	0.34
Age	0.14	0.14	0.99	0.32
**"Life's Too Short"**				
Neural similarity	0.22	0.22	1.00	0.32
Handedness	0.07	0.06	1.16	0.25
Nationality***	0.61	0.16	3.89	0.00011
Gender**	0.37	0.12	3.07	0.0021
Ethnicity	0.08	0.09	0.86	0.39
Age	0.14	0.14	0.97	0.33
**Sentimental/'Cute' videos**				
**"All I Want" (music video)**				
Neural similarity	-0.30	0.15	-1.96	0.051
Handedness	0.08	0.06	1.28	0.20
Nationality***	0.58	0.16	3.72	0.00021
Gender**	0.36	0.12	3.02	0.0026
Ethnicity	0.09	0.10	0.86	0.35
Age	0.12	0.14	0.86	0.39
**Wedding film**				
Neural similarity	0.02	0.22	0.07	0.94
Handedness	0.07	0.06	1.21	0.23
Nationality***	0.59	0.16	3.81	0.00015
Gender**	0.36	0.12	3.04	0.0024
Ethnicity	0.08	0.10	0.89	0.37
Age	0.14	0.14	0.97	0.33
**Baby sloth sanctuary (documentary clip)**				
Neural similarity	-0.07	0.22	-0.31	0.75
Handedness	0.07	0.06	1.22	0.22
Nationality***	0.59	0.16	3.86	0.00012
Gender**	0.37	0.12	3.09	0.0021
Ethnicity	0.09	0.10	0.91	0.36

Age	0.14	0.14	0.97	0.33
**Debates/Social issues**				
**“Ban College Football” (debate)**				
Neural similarity	-0.44	0.24	-1.83	0.068
Handedness	0.06	0.05	1.07	0.28
Nationality***	0.58	0.15	3.83	0.00013
Gender**	0.36	0.12	2.95	0.0033
Ethnicity	0.08	0.09	0.92	0.36
Age	0.13	0.14	0.94	0.35
**Crossfire (political clip)**				
Neural similarity	-0.15	0.24	-0.62	0.54
Handedness	0.07	0.06	1.29	0.20
Nationality***	0.59	0.15	3.86	0.00012
Gender**	0.36	0.12	3.07	0.0022
Ethnicity	0.08	0.09	0.90	0.37
Age	0.14	0.14	1.01	0.31
**“Food Inc.” (documentary clip)**				
Neural similarity	-0.26	0.24	-1.11	0.27
Handedness	0.06	0.06	1.05	0.30
Nationality***	0.59	0.15	3.85	0.00013
Gender**	0.37	0.12	3.11	0.0019
Ethnicity	0.08	0.10	0.86	0.39
Age	0.14	0.14	1.01	0.31
**Miscellaneous (videos selected because they might be interesting to some people but not others)**				
**Google Glass review**				
Neural similarity	-0.16	0.18	-0.94	0.35
Handedness	0.07	0.06	1.32	0.19
Nationality***	0.59	0.15	3.85	0.00013
Gender**	0.37	0.12	3.06	0.0023
Ethnicity	0.08	0.09	0.90	0.37
Age	0.14	0.14	0.99	0.32
**Soccer match**				
Neural similarity**	-0.53	0.21	-2.58	0.010
Handedness	0.10	0.06	1.64	0.10
Nationality***	0.58	0.15	3.83	0.00014
Gender**	0.36	0.12	3.09	0.0021
Ethnicity	0.09	0.09	0.97	0.33
Age	0.11	0.14	0.82	0.41
**Scientific demonstration (washcloth wrung out in space)**				
Neural similarity	-0.05	0.27	-0.21	0.84
Handedness	0.07	0.06	1.25	0.21
Nationality***	0.59	0.15	3.77	0.00018
Gender**	0.36	0.12	3.04	0.0025
Ethnicity	0.08	0.10	0.89	0.37
Age	0.14	0.14	0.97	0.33
**“An Astronaut’s View of Earth”**				
Neural similarity***	-0.62	0.19	-3.30	0.0010
Handedness	0.09	0.06	1.43	0.15
Nationality***	0.60	0.15	4.10	0.000045
Gender**	0.39	0.12	3.17	0.0016
Ethnicity	0.10	0.10	1.07	0.29
Age	0.14	0.14	0.99	0.32
***$p \leq .001$, **$p \leq .01$, *$p \leq .05$ (two-tailed)				

Supplementary Note 1

Defining social distance based on both reciprocated and unreciprocated social ties. Our main analyses defined social ties based only on reciprocated ties. We reasoned that some unreciprocated ties may be the result of some participants tending to nominate large numbers of classmates as friends (out-degree ranged from 2 to 146), and that mutually reported ties were most likely to correspond to meaningful friendships. The same pattern of results as is reported in the main text was achieved when defining social distance based on both reciprocated and unreciprocated ties. An ordered logistic regression model revealed a significant effect of neural similarity (ordered logistic regression: β = -0.26, *SE* = 0.12, *p* = .029; *N* = 861 dyads) on social distance that was comparable in magnitude to our main results: holding other covariates constant, compared to a dyad at the mean level of neural similarity and at any given level of social distance, a dyad one standard deviation more similar is 23% more likely to have social distance that is one unit shorter. Of the control variables also included in the model, dyadic dissimilarities in gender (ordered logistic regression: β = 0.37, *SE* = 0.10, *p* = .0003; *N* = 861 dyads), nationality (ordered logistic regression: β = 0.78, *SE* = 0.15, *p* = 1.2×10^{-7}; *N* = 861 dyads), and ethnicity ordered logistic regression: β = 0.26, *SE* = 0.053, *p* = 7.6×10^{-7}; *N* = 861 dyads) were also related to social distance, whereas age (ordered logistic regression: β = 0.0255, *SE* = 0.124, *p* = .84; *N* = 861 dyads) and handedness (ordered logistic regression: β = 0.18, *SE* = 0.12, *p* = .14; *N* = 861 dyads) were not. A likelihood ratio test indicated that neural similarity added significant predictive power, above and beyond observable demographic similarity, $\chi^2(1) = 9.61$, *p* = .0019.

Supplementary Note 2

Testing whether neural similarity is associated with social network proximity without normalizing neural similarities within brain region. Prior to conducting the analyses reported in the main text, correlation coefficients were z-scored for each brain region across dyads in order to have a mean of 0 and a standard deviation of 1. This normalization step was performed to account for the fact that brain regions would likely vary in the extent to which they would become coupled across participants overall, as well as in the extent to which that coupling would vary across dyads, and we sought to characterize how similar neural responses were for a given pair of participants for a given brain region, relative to the similarity of all dyads' responses for that brain region. We also repeated our main analyses without z-scoring the Pearson correlation coefficients, and found the same pattern of results that is reported in the main text. Specifically, in an ordered logistic regression using social distance as the dependent variable and the dissimilarities in control variables (handedness, ethnicity, nationality, age, gender) and weighted (by ROI volume) average neural similarity (based on the Pearson correlation coefficients between preprocessed time series for each brain region for each unique pair of participants) as predictor variables, there was a significant effect of neural similarity on social distance (ordered logistic regression: $\beta = -0.232$, $SE = 0.108$, $p = .03$; $N = 861$ dyads) similar to the results reported in the main text. There was also a significant relationship between social distance and dissimilarity in gender (ordered logistic regression: $\beta = 0.387$, $SE = 0.122$, $p = .002$; $N = 861$ dyads) and nationality (ordered logistic regression: $\beta = 0.564$, $SE = 0.149$, $p = .0002$; $N = 861$ dyads). As in the analyses reported in the main text, dissimilarity in ethnicity (ordered logistic regression: $\beta = 0.092$, $SE = 0.094$, $p = .33$; $N = 861$ dyads), age (ordered logistic regression: $\beta = 0.130$, $SE = 0.137$, $p = .34$; $N = 861$ dyads), and handedness (ordered logistic

regression: $\beta = 0.085$, $SE = 0.060$, $p = .16$; $N = 861$ dyads) were not predictive of social distance. A likelihood ratio test comparing the model described above to a model that did not include a term corresponding to neural similarity indicated that (un-normalized) average overall neural similarity added additional predictive power, above and beyond similarity in terms of the observed demographic variables, $\chi^2(1) = 11.987$, $p = .0005$.

Supplementary Note 3

Testing whether neural similarity is associated with social network proximity without weighting neural similarities by brain region volume. The analyses reported in the main text that probe the relationship between social network proximity and overall neural similarity, collapsed across brain regions, involve weighting each region of interest (ROI) by volume prior to averaging. As noted in the main text, a similar pattern of results was obtained when weighting every ROI equivalently, irrespective of its volume. In an ordered logistic regression using social distance as the dependent variable and the dissimilarities in control variables (handedness, ethnicity, nationality, age, gender), unweighted average neural similarity as predictor variables, and multi-way clustering to account for the non-independence of dyadic observations, there was a marginally significant effect of neural similarity on social distance (ordered logistic regression: $\beta = -0.195$, $SE = 0.104$, $p = .06$; $N = 861$ dyads), and a significant relationship between social distance and dissimilarity in gender (ordered logistic regression: $\beta = 0.381$, $SE = 0.122$, $p = .002$; $N = 861$ dyads) and nationality (ordered logistic regression: $\beta = 0.566$, $SE = 0.151$, $p = .0002$; $N = 861$ dyads). Consistent with the analyses reported in the main text, dissimilarity in ethnicity (ordered logistic regression: $\beta = 0.094$, $SE = 0.095$, $p = .32$; $N = 861$ dyads), age (ordered logistic regression: $\beta = 0.132$, $SE = 0.137$, $p = .34$; $N = 861$ dyads), and handedness (ordered logistic regression: $\beta = 0.082$, $SE = 0.059$, $p = .17$; $N = 861$ dyads) were not predictive of social distance. A likelihood ratio test comparing the model described above to a model that did not include a term corresponding to neural similarity indicated that (unweighted) average overall neural similarity added additional predictive power, above and beyond similarity in terms of the observed demographic variables, $\chi^2(1) = 8.477$, $p = .0036$.

Supplementary Note 4

Accounting for participants' previous familiarity with videos. As reported in Supplementary Table 1 and the Methods section, post-scan interviews indicated that the majority of participants had no previous familiarity with the video stimuli used in the neuroimaging study. Five of the 14 videos had been seen by both members of one or more dyads (please see the 'Prior familiarity with stimuli' sub-section of the Methods section for further details). After excluding any dyads whose members had both seen the same clips prior to participating in the study, the effect of neural similarity on social distance remained significant (ordered logistic regression: $\beta = -0.218$, $SE = 0.107$, $p = .042$; $N = 848$ dyads) in our main ordered logistic regression analysis.

Supplementary Note 5

Permutation testing based on network randomization. We also performed permutation testing of the data to supplement the analyses described in the main text. We adopted the topological clustering methods employed by Christakis and Fowler[1] to test if there was a greater degree of clustering of particular neural response patterns than would be expected based on chance (i.e., if there was exceptionally high neural similarity among individuals close together in the social network). This method entailed iteratively computing the neural similarity between all individuals in the network in 1,000 randomly generated datasets in which the topology of the social network and the prevalence of particular neural response patterns were held constant while the assignment of neural data to individuals was randomly shuffled.

More specifically, a distribution of Pearson correlation coefficients corresponding to the null hypothesis that no relationship exists between social distance and neural similarity was obtained by randomly shuffling the neural time series data among participants 1,000 times, then computing the weighted (by ROI volume, as described in the main text) average neural similarity for dyads in each social distance category for each of the 1,000 randomly generated permutations of the dataset. Each participant's neural time series data consists of an 80 (brain regions) x 1,010 (time points) matrix – i.e., a set of 80 time series, each consisting of 1,010 time points. These neural time series datasets were randomly shuffled among the 42 fMRI study participants 1,000 times while keeping the social network data characterizing connections between participants constant. The magnitude of the weighted average neural similarity for each social distance category within each of the randomly permuted datasets was compared to that of the original, non-permuted data.

Results of these permutation tests revealed a similar pattern of results to those described in the main text and are illustrated in Fig. 6. Distance 1 dyads' ($N = 63$) neural response time series were, on average, exceptionally more similar to one another than would be expected based on chance, $p = .03$. There was a non-significant trend such that distance 2 dyads ($N = 286$) were marginally more similar to one another than would be expected based on chance, $p = .06$. Distance 3 dyads ($N = 412$) were exceptionally less similar to one another than would be expected based on chance, $p = .003$. Distance 4 dyads ($N = 100$) were neither more or less similar to one another than would be expected based on chance, $p = .5$. We note that that the fact that distance 3 dyads were significantly less similar to one another than would be expected based on chance alone does not imply that members of these dyads had anti-correlated neural response time series. Rather, members of distance 3 dyads were characterized by neural response similarities that were smaller in magnitude than would be expected if there were no relationship between neural response similarity and proximity in the social network.

Supplementary Methods

Deviation coding of estimates in Figure 4d. There are many ways to code categorical variables for regression. Conventional dummy coding (where each observation gets a value of 1 for its category and a 0 for other categories) is useful for comparing all other categories against a single "baseline" category. Deviation coding is more appropriate for comparing each category against the overall mean of the sample. In this case, deviation coding measures, for each social distance, a point estimate and confidence interval of the difference in neural similarity from the average of the other social distance categories, after partialing out the effects of control variables (age, nationality, ethnicity, gender, and handedness). To make these estimates, we first calculated deviation-coded dummy variables corresponding to each value of social distance, 2 through 4. Unlike conventional coding of dummy variables, deviation-coded dummy variables take a value of -1 when social distance is equal to one. These deviation-coded dummy variables are then entered, together with variables describing inter-subject differences in demographic variables and handedness, into an ordinary least squares regression model of the standardized, weighted neural similarity measure. As in our primary analyses, estimates were clustered simultaneously on both members of each dyad. The point estimate and confidence interval for distance one dyads were estimated from the intercept; point estimates and confidences intervals for dyads at distances two through four were estimated from their respective deviation-coded variables.

Supplementary References

1. Christakis, N. A. & Fowler, J. H. Social contagion theory: Examining dynamic social networks and human behavior. *Stat. Med.* **32,** 556–77 (2013).
